# Low concentrations of grape seed extract maintain osteoblast morphology, cell adhesion, and mineralization

**DOI:** 10.1590/0103-6440202304987

**Published:** 2023-05-15

**Authors:** Maria Carolina Coelho, Viviann Ruocco Vetucci, Roger Rodrigo Fernandes, Paula Katherine Vargas Sanchez, Selma Siessere, Karina Fittipaldi Bombonato-Prado

**Affiliations:** 1 Bone Research Lab, Department of Basic and Oral Biology, School of Dentistry of Ribeirão Preto, University of São Paulo, Ribeirão Preto, SP, Brazil; 2 Department of Oral and Maxillofacial Surgery and Periodontology, School of Dentistry of Ribeirão Preto, University of São Paulo, Ribeirão Preto, SP, Brazil; 3 Unit of Basic Oral Investigation-UIBO, School of Dentistry, Universidad El Bosque, Bogotá, Colombia.

**Keywords:** grape seed extract, cell morphology, osteoblasts, mineralization

## Abstract

The increase in life expectancy has led to a higher incidence of osteoporosis, characterized by an imbalance in bone remodeling. Several drugs are used for its treatment, but most promote undesirable side effects. The present investigation evaluated the effects of two low concentrations of grape seed extract (GSE) rich in proanthocyanidins on MC3T3-E1 osteoblastic cells. The cells were cultured in an osteogenic medium and divided into control (C), 0.1 µg/mL GSE (GSE_0.1_), and 1.0 µg/mL GSE (GSE_1.0_) groups to evaluate cell morphology, adhesion, and proliferation, in situ alkaline phosphatase (ALP) detection, mineralization and immunolocalization of osteopontin (OPN). The data obtained were analyzed by statistical tests for a significance of 5%. Cell morphology was maintained with both GSE concentrations, whereas cell adhesion significantly increased within three days in all groups. Cell proliferation increased significantly at seven days of culture, followed by a significant decrease in all experimental periods, with no statistical difference among them. *In situ* detection of ALP and mineralization increased with time, but within each period, no statistical differences among groups were observed. The expression of osteopontin was distributed regularly with more intensity after 24 hours in the GSE_0.1_ group. After three days, OPN expression was more intense in the control group, followed by GSE_0.1_ and GSE_1.0_ groups. Data obtained suggest that low concentrations of GSE do not affect the morphology and may stimulate the functional activity of osteoblastic cells.

## Resumo

O aumento da expectativa de vida tem levado a uma maior incidência de osteoporose, caracterizada por um desequilíbrio na remodelação óssea. Vários medicamentos são utilizados para o seu tratamento, contudo, a maioria promove efeitos colaterais indesejáveis. A presente investigação avaliou os efeitos de duas baixas concentrações de extrato de semente de uva (GSE) rico em proantocianidinas em células osteoblásticas MC3T3-E1. As células foram cultivadas em meio osteogênico e divididas em grupos controle (C), 0,1 µg/mL de GSE (GSE_0.1_) e 1,0 µg/mL de GSE (GSE_1.0_) para avaliar morfologia, adesão e proliferação celular, detecção *in situ* de fosfatase alcalina (ALP), mineralização e imunolocalização da proteína osteopontina (OPN). Os dados obtidos foram analisados por testes estatísticos para um nível de significância de 5%. A proliferação celular aumentou significativamente aos sete dias de cultura, seguido de uma diminuição significativa em todos os períodos experimentais, sem diferença estatística entre eles. A detecção *in situ* de ALP e mineralização aumentou com o tempo, mas dentro de cada período não foram observadas diferenças estatísticas entre os grupos. A morfologia celular foi mantida com ambas as concentrações de GSE, enquanto a adesão celular aumentou significativamente aos três dias em todos os grupos. A expressão de osteopontina distribuiu-se regularmente com maior intensidade após 24 horas no grupo GSE_0.1_. Após três dias, a expressão de OPN foi mais intensa no grupo controle, seguida pelos grupos GSE_0.1_ e GSE_1.0_. Os dados obtidos sugerem que baixas concentrações de GSE não afetam a morfologia e podem estimular a atividade funcional das células osteoblásticas.

## Introduction

Bone tissue has been the target of numerous investigations over time, mainly regarding its resorption as a consequence of diseases such as osteoporosis. Bone is a calcified tissue composed of 60% inorganic component (hydroxyapatite), 10% water, and 30% organic component (proteins), involving mainly cells such as osteoblasts, osteocytes, osteoprogenitor cells, and osteoclasts ^1^.

Osteoporosis is characterized by the loss of bone tissue microarchitecture with the reduction in the number of osteoblasts and loss of calcification of the pre-existing extracellular matrix. The treatment and prevention of osteoporosis can be carried out by drugs such as oral bisphosphonates ^2^, which can lead to gastrointestinal disturbances as well as osteonecrosis of the jaws ^3^.

Given these facts, an increase in the search for alternative natural substances for the treatment of osteoporosis that promote fewer side effects has been observed. Earlier reports show the effect of several antioxidant substances, with an increase in the differentiation of osteoblasts and cell proliferation in MC3T3-E1 lineage when exposed to the flavonoid and carotenoid extract of *Cissus quadrangularis*, a plant from the same grape family ^4^, as well as when exposed to catechins present in green tea extract, with suppression of bone resorption ^5^.

Grape seed extract (GSE) is rich in polyphenols, with its main active compound being proanthocyanidin, with pharmacological and therapeutic potential due to its antioxidant, immunostimulating, anticarcinogenic, antiallergic activity, and anti-inflammatory effects ^6^. In addition, proanthocyanidin protects cells like MC3T3-E1 from oxidation induced by mitochondrial dysfunction and reduces osteoblast apoptosis ^2^. In a study by Toker *et al*. ^7^, grape seed extract reduced the loss of alveolar bone by reducing the mediators MMP-8 and HIF-1α. The grape seed extract is also capable of reducing the differentiation of osteoclasts, cells responsible for bone resorption ^8^. Given the above, it can be inferred that grape seed extract, rich in proanthocyanidin, can induce proliferation and increase the protein activity of osteoblast cells (MC3T3-E1). The present work hypothesizes that low concentrations of the grape seed extract can stimulate osteoblastic cells, without damaging their morphology and functional activity.

## Materials and methods

### Cell culture

MC3T3-E1 cells (sub-clone 14, ATCC, Manassas, VA, USA) were grown in 75 cm^3^ culture bottles with 10 mL of α-MEM culture medium, 10% fetal bovine serum, 2.75 mL of penicillin-streptomycin, 50 µg of ascorbic acid and 2mM beta-glycerolphosphate ^9^ ^,^ ^10^. The cells were kept at 37 °C throughout the entire culture in a humidified environment with 5% CO2 and 95% atmospheric air, and the culture medium was changed every two days. Following confluence, the cells were transferred from the culture flasks to 24-well culture plates by being treated with 1 mM EDTA (Gibco, USA) and 0.25% trypsin (Gibco, USA). The following groups of cells were created: control (GSE was not added), the addition of 0.1 µ/mL GSE, and the addition of 1.0 µ/mL GSE. For each in vitro experiment, there were seeded 5 wells per group for each assessed period.

### In vitro administration of grape seed extract (GSE)

In the current study, a grape seed extract with at least 90% polyphenols was used (Meganatural Seed-BP, Healthy Origins, USA). The concentrations of 0.1 and 1 µ/mL GSE ^8^ were dissolved in the culture medium from a stock solution, remaining in contact with the cells throughout cell culture and being replenished with each media change.

### Cell morphology and OPN expression

Cells were cultivated on glass coverslips for cell morphology analysis and immunolocalization of the osteopontin (OPN) protein. After 24 and 3 days of culture, the cells were fixed in 4% paraformaldehyde in 0.1 M phosphate buffer (PB), pH 7.2, for 10 minutes at room temperature. After that, the cells underwent regular indirect immunofluorescence processing ^11^. After blocking for 30 minutes with 5% skimmed milk in PB, permeabilization was carried out using 0.5% Triton X-100 solution in PB for 10 minutes. A secondary antibody labeled with Alexa Fluor 594 (Molecular Probes, USA), diluted to 1:200, was then incubated for 50 minutes after the main monoclonal antibody for OPN (MPIIIB10, 1:800 dilution, Developmental Studies Hybridoma Bank, USA) had been incubated for an hour. Phalloidin coupled with Alexa Fluor 488 (1:200) and DAPI were utilized to visualize cell boundaries and cell nuclei attached to the coverslip. As a negative control, primary PB antibodies were swapped out. The markings were examined using a Leica fluorescence microscope after placing a glass coverslip on the discs with an anti-fade mounting medium (Prolong, Molecular Probes, Germany).

### Cell adhesion and proliferation

After 24 hours and 3 days of culture, cell adhesion was evaluated by nuclei counting through DAPI (4′,6-diamidino-2-phenylindole, Molecular Probes, USA) staining and observation in a fluorescence microscope (Leica DM4000B, Germany). At 3, 7, and 10 days of culture, proliferation was assessed by the MTT colorimetric assay {[3- (4,5-dimethylthiazol-2-yl) -2,5-diphenyltetrazolium]} (Sigma). After removing the culture medium, the cells were incubated with MTT + MTS (supplemented total culture medium) solution for 4 hours at 37 ºC, in a humidified atmosphere containing 5% of CO_2_ and 95% of atmospheric air. After this period, the culture medium was removed from the wells, and then 1 mL of acid isopropanol solution (Merck, Germany) was added to each well under agitation for 5 minutes, for complete solubilization of the precipitate formed. Following, 150 μL aliquots were removed from the wells and transferred to a 96-well plate for reading on a spectrophotometer (μQuant, Biotek Instruments Inc., USA) at a wavelength of 570 nm.

### Alkaline phosphatase detection assay

The *in situ* detection of enzyme alkaline phosphatase was performed at 3, 7, and 10 days. After removing the culture medium, the wells were washed twice with PBS (Phosphate buffered saline) solution heated to 37 °C. Then, three hundred and twenty milligrams of the Triz reagent (Sigma) were dissolved in 20 mL of deionized water and, added, 7 mg of the Fast Red reagent (Sigma). 2 mL of this solution was discarded and 8 mg of naphthol (Sigma) diluted in 2 mL of dimethylformamide (Merck) were added to form the working solution. One milliliter of this solution was added to each well. The plate was then taken to the incubator in a humidified atmosphere at 37 °C with 5% of CO_2_ for 30 minutes. The solution was then taken out of the wells, and the plate was allowed to dry at room temperature before being subjected to qualitative and quantitative examination using Image J.

### Quantification and detection of mineralized nodules

The detection of the mineralized matrix was evaluated at the end of the 17 days of the experiment. After removing the culture medium, the wells were washed three times with PBS (GibcoTM, USA) heated to 37 ºC, and 2 mL of 10% formalin was added for fixation and maintained at 4 °C for 24 hours. After that time, formalin was removed and the wells were dehydrated at room temperature in increasing series of alcohols (30%, 50%, 70%, and 100%) for a period of 1 hour for each alcoholic graduation. After drying, the wells were stained with 2% alizarin red pH 4.2 (Sigma) for 10 minutes, and the mineralized areas rich in calcium were evidenced by the red color. The quantification was performed according to Gregory *et al*. ^12^, where 280 μL of 10% acetic acid (Labsynth, Brazil) were added to each well and the plates were left under gentle agitation for 30 minutes. The cell layer was then scraped with the aid of a tip and the solution was transferred to 1.5 mL Eppendorf tubes, heated to 85 ºC for 10 minutes, and transferred to the ice for 5 minutes. The tubes were centrifuged at 13.000 *rpm* for 20 minutes. A volume of 150 μL of the supernatant was transferred to a 96-well plate (Corning) and 40 μL of 10% ammonium hydroxide (Quimibras, Brazil). The reading was performed on a spectrophotometer (Bio-Tek) at a wavelength of 405 nm.

### Statistical analysis

The obtained data were evaluated for data normality. The analysis of variance (ANOVA) was then used to examine the parametric data, and a post-test for multiple comparisons between groups was conducted. GraphPad Prism 5.0 software was used, and the significance level was set at 5% (GraphPad Software, USA).

## Results

### Cell morphology and OPN expression

**Figure 1:**
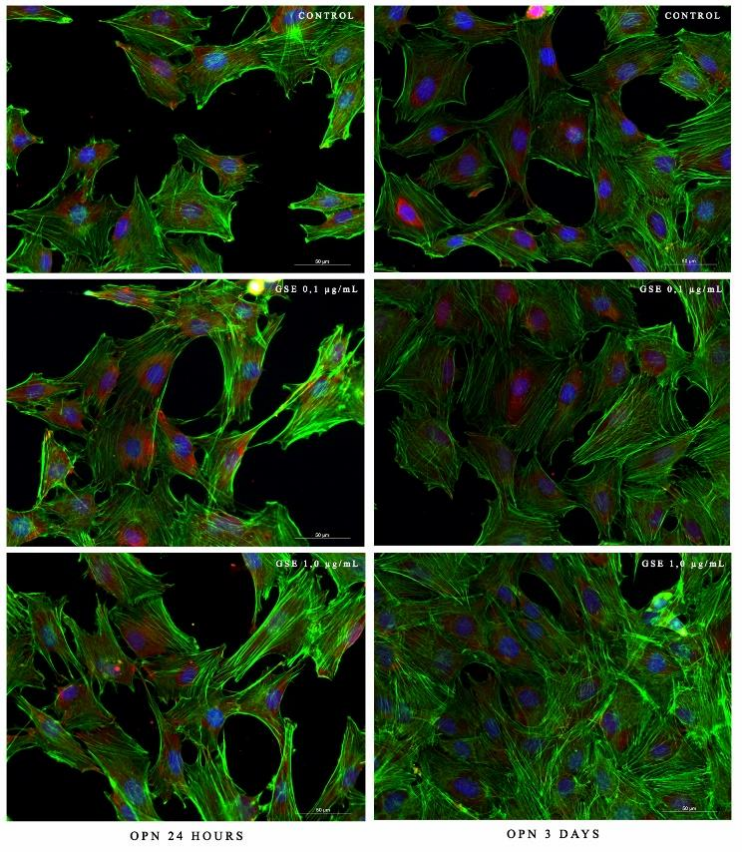
Cell morphology and immunolocalization of osteopontin (OPN) in MC3T3-E1 osteoblastic cells after 24 hours and 3 days of culture. Bar scale = 50 µm. Green = cytoskeletal; red = osteopontin; blue = cell nucleus.

The morphology and expression of OPN protein were observed after 24 hours and 3 days of cell culture. The control and treated cells cultured for 24 hours and 3 days were well spread to the polystyrene slide, exhibiting normal cytoskeletal architecture represented by actin staining (green staining). All cells showed intact nuclei without signs of apoptosis (blue staining). The expression of the osteopontin (red staining) was distributed regularly in the cytoplasm adjacent to the nucleus with more intensity after 24 hours of culture in the groups GSE_0.1_, followed by control and GSE_1.0_. After three days, the expression of OPN was more intense in the control group, followed by GSE_0.1_ and GSE_1.0_ (Figure 1).

### Cell adhesion

**Figure 2:**
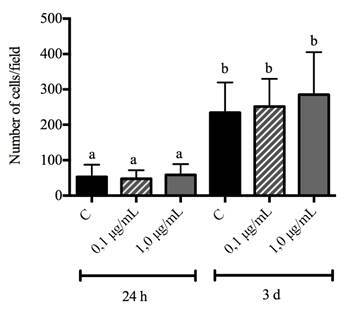
Adhesion of MC3T3-E1 osteoblastic cells after 24 hours and 3 days. Different letters mean statistical difference for p<0.05. ANOVA statistical test was performed.

After 24 hours and 3 days of cell culture, immunofluorescence assays were performed with nuclei staining (DAPI) and cell counting. The results showed that cell adhesion was significantly increased in three days (p<0.001), with no differences between the control and cells that received both concentrations of GSE in the culture medium (Figure 2).

### Cell proliferation

**Figure 3:**
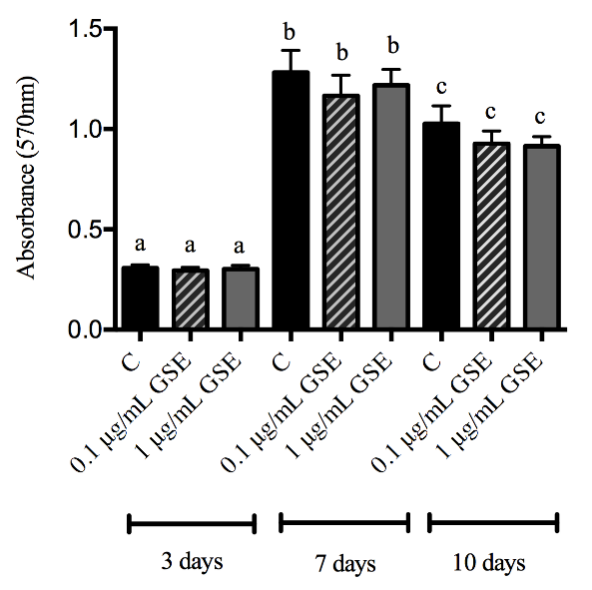
Proliferation of MC3T3-E1 osteoblastic cells, divided into control (C), GSE_0.1_, and GSE_1.0_ groups cultured for 3, 7, and 10 days. Different letters mean statistical difference for p<0.05. ANOVA statistical test was performed.

Figure 3 shows that cell proliferation increased significantly with a peak at seven days of culture, followed by a significant decrease in all experimental periods (p<0.01), with no statistical difference among them.

### Alkaline phosphatase assay

**Figure 4:**
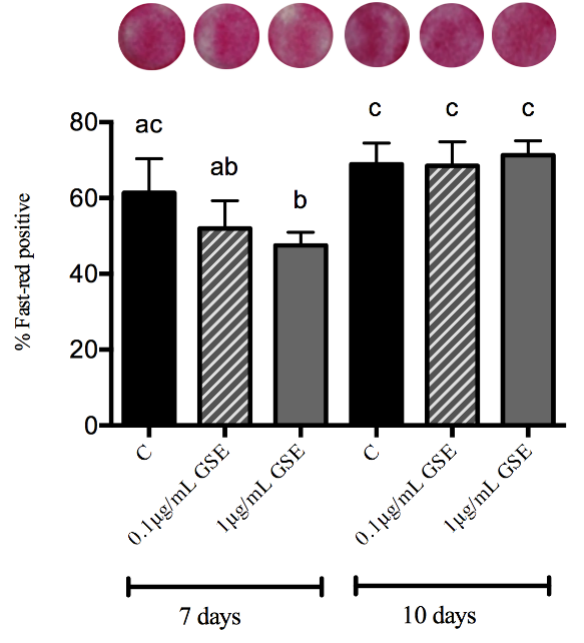
Alkaline phosphatase (ALP) *in situ* detection in MC3T3-E1 osteoblastic cells after 7 and 10 days of culture in control (C), GSE_0.1_, and GSE_1.0_ groups. Different letters mean statistical difference for p<0.05. ANOVA statistical test was performed.

Data presented in Figure 4 show that ALP (alkaline phosphatase) *in situ* detection significantly increased along the days of culture, with no statistical difference between the groups that received both doses of GSE and the control.

### Quantification and detection of mineralized nodules

**Figure 5:**
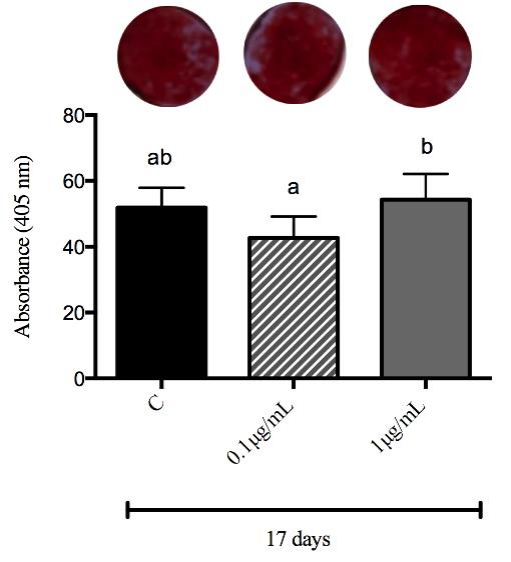
Mineralization after 17 days in MC3T3-E1 osteoblastic cell culture, divided into control (C), GSE_0.1_, and GSE_1.0_ groups. Different letters mean statistical difference for p<0.05. ANOVA statistical test was performed.

Figure 5 shows a significant amount of mineralized nodules at 17 days of culture in all experimental periods, with no statistical differences between the control and the groups that received both GSE concentrations. Nevertheless, the group that received GSE_1.0_ had a significant increase in mineralization when compared to the GSE_0.1_ group (p<0.05).

## Discussion

Osteoporosis is a very common chronic condition that primarily affects postmenopausal women. It is characterized by a breakdown of equilibrium between bone resorption and apposition, which results in a decrease in bone density ^13^. Although there are a number of treatments including hormone replacement, osteoclastic activation-related antibodies, and bisphosphonates, their use is constrained by unfavorable side effects and maximal usage duration ^14^ ^,^ ^15^.

As a result, the search for natural components that may be utilized in place of traditional therapies, but have no side effects, has significantly increased. According to epidemiological studies, women's enhanced bone mineral density is closely correlated with flavonoid intake ^16^. Grape seed extract (GSE) is a powerful antioxidant with a polyphenolic structure and a broad spectrum of biological activity ^7^, in addition to being widely available commercially in capsules or compounding pharmacies. In order to test the hypothesis that GSE does not cause cell toxicity, our study examined the effects of GSE at two low doses on the morphology and functional activity of osteoblastic cells in standard culture conditions.

Our hypothesis was confirmed regarding the maintenance of cell morphology and organization of cytoplasm/nuclei, suggesting that GSE exposition was not harmful to the cells in both low concentrations. Kwak *et al.* ^17^ also observed that grape seed extract does not show cytotoxicity in bone marrow cells. The intense osteopontin expression observed in the cytoplasm adjacent to the nucleus in the majority of cells of the evaluated groups suggests its role in the main biological activities of bone cell metabolism such as cell adhesion and proliferation, which were similar in cells with and without GSE in culture medium. According to Si *et al*. ^18^, osteopontin is involved in activities related to the proliferation, migration, and adhesion of different bone cells, including bone marrow mesenchymal stem cells, hematopoietic stem cells, osteoclasts, and osteoblasts, as well as its relationship with diseases such as osteoporosis, rheumatoid arthritis, and osteosarcoma. According to Vimalraj ^19^, the alkaline phosphatase protein (ALP) is expressed in large amounts in mineralized tissues, playing an essential role in hard tissue formation and bone matrix mineralization and, in our study, GSE did not interfere with its production and release, both in early as late culture periods, contributing to mineralization.

There are few studies involving the effects of GSE on osteoblastic functional activity through in vitro experiments in the literature. A report published by Torre *et al*. ^20^ showed that grape extract (skin, pulp, and seeds) induces increased expression of genes related to osteoblastic differentiation in bone marrow mesenchymal cells. Dai *et al*. ^21^ also demonstrated that resveratrol (a non-flavonoid phenolic compound found in grapes) stimulates osteoblastic proliferation and differentiation of bone marrow mesenchymal cells by activating the ERK1 / 2 / pathway. Studies in this area carried out with the MC3T3-E1 cells were published by Zhang *et al.* ^2^, but with oxidative stress induction by hydrogen peroxide. These authors observed that grape seed extract inhibited cell apoptosis mediated by p53. Oral GSE delivery enhances osseointegration, bone healing, and mechanical strength following fractures in conditions of estrogen deficiency, such as menopause, according to in vivo research with animal models ^22^ ^,^ ^23^.

The low concentrations used in the study were selected due to the fact that high levels of GSE can affect cells by inhibiting their proliferation, e.g., inhibition of oral cancer Ca9-22 cells ^24^. According to these authors, high GSE concentrations (50-400 μg/mL) cause a reduction in cell proliferation, whereas low concentrations (1-10 μg/mL) do not have the same effect. The same author suggests that this is due to the generation of reactive oxygen species (ROS) and mitochondrial depolarization. Therefore, the obtained results are encouraging and advances can be achieved concerning the use of GSE, which must be investigated with different parameters*.*

As an*in vitro*study, it has its limitations and further studies should be carried out to evaluate the effect of GSE on*in vivo*applications in experimental models of osteoporosis since the GSE did not affect the morphology and did not inhibit osteoblastic cell function associated with increased bone matrix deposition. If more studies are carried out, GSE may become a promising alternative herbal treatment for osteoporosis.
